# Editorial: Frontiers in bacterial quorum sensing research

**DOI:** 10.3389/fcimb.2022.999388

**Published:** 2022-08-31

**Authors:** Lin Chen, Lina Ku, Mingkai Li

**Affiliations:** ^1^ Key Laboratory of Resource Biology and Biotechnology in Western China, Ministry of Education, Northwest University, Xi’an, China; ^2^ Department of Pharmacology, School of Pharmacy, the Fourth Military Medical University, Xi’an, China

**Keywords:** bacteria, quorum, resistance, biofilm, virulence

Recent discoveries expand our knowledge of quorum sensing (QS) mediated communication systems, and demonstrate that QS are widespread in the microbial world including bacteria, fungi and virus (Whiteley et al., 2017; Tian et al., 2021). QS is a process of cell-to-cell communication, this complex signaling network allows large populations of microbial cells to exhibit as a multicellular behavioral pattern in response to changing environment conditions (Abisado et al., 2018). With the increase in population density, the QS signals accumulate in the extracellular environment and once the concentration reaches a threshold, it will be sensed and detected by microbial cells, then the genes expression is coordinated (Novick and Geisinger, 2008). The cell density dependent signaling system enables microbial cells to behave as multi-cellular organisms in response to environmental cues during different microbial behaviors like morphogenesis, pathogenesis, competition, and mutualistic coordination, which microbial use as a survival strategy in nature environments.

QS plays a vital role in many processes, and numerous milestone discoveries reveal the complexity of its metabolic and signaling networks. Understanding QS related mechanisms is a key step in many applications including anti-pathogen, anti-biofilm, sustainable microbial fuel, bioremediation and agriculture (Skandamis and Nychas, 2012; Pan et al., 2020). Therefore, discoveries of QS and QS inhibitor (QSI) opened a door to combating the microbial pathogenesis, reducing the formation of bacterial biofilm, and controlling bioremediation (Kalia et al., 2019). As a result, current topic focused on advances in bacterial quorum sensing system and quenching.

Two manuscripts go in-depth in the study of the role of QS in two opportunistic pathogens, *Acinetobacter baumannii* (*A. baumannii*) and *Stenotrophomonas maltophilia* (*S. maltophilia*), belonging to the γ-proteobacteria class. Sun et al. reported the contribution of the *abaI*/*abaR* QS system in *A. baumannii*, the most troublesome pathogen for healthcare institutions globally. Not surprisingly, the *abaI*/*abaR* system plays an essential role in drug resistance, biofilm formation, and virulence production in *A. baumannii*. What call for special attention is that destructing the receptor *abaR* has the converse result with interdicting the acyl homoserine lactone synthesis enzyme *abaI*. Deletion of *abaR* enhances the activity of immune evasion, which is verified by injection of bacteria into *Galleria mellonella* larvae. This is probably due to the significant overexpression of multi- transport genes including carbohydrate transport, amino acid transport, lipid transport and their related metabolism genes. Overall, these results provide a new insight into the *abaI*/*abaR* QS system effects on pathogenicity in *A. baumannii*. In *S. maltophilia*, Yero et al. established a link between diffusible signal factor DSF-based QS system and the virulence/resistance phenotypes, confirmed in a collection of 78 geographically and genetically diverse clinical strains. Two cluster variants, *rpf*-1 and *rpf*-2, are responsible for its synthesis and perception of the main QS signaling molecule in *S. maltophilia*. The former is resistant to the β-lactam antibiotics ceftazidime and ticarcillin, while the latter exhibits higher resistance to colistin. Strains of variant *rpf*-2 also show significantly more virulent to *G. mellonella* larvae than those of *rpf*-1, most likely due to an increased ability of bacterial biofilm formation.

Although the majority of studies focused on the pathogenicity of bacterial QS, the beneficial properties of soil microorganisms QS in plant growth promoting rhizobacteria (PGPR) are uncovered recently. The study conducted by Jung et al. revealed the influence of the QS dependent genes of *Serratia fonticola* GS2 on potential plant growth promoting (PGP) activities. Based on genomic, molecular and phenotypic experimental data, they confirm the biological functionality of QS auto-inducer (*gloI*) and receptor (*gloR*) on PGP activities. *In vivo* experiments confirm that plants treated with wild type stains had significantly higher growth rates than plants treated with the QS deletion mutants, which is probably due to the QS dependent influence on PGP activities including indole-3-acetic acid (IAA) production, 1-aminocyclopropane-1-carboxylate (ACC) deaminase activity, and biofilm formation.

Virulence expression, biofilm formation and antibiotic resistance are the typical processes regulated by QS. Therefore, QSI seems a promising approach to the related challenges in a large variety of applications including human healthcare, food industry, environment and agriculture (Mukherjee and Bassler, 2019). Contributing to this topic in the context of the implication of QSI on the mixed bacterial biofilms, Shukla et al. evaluated the inhibitory potential of the purified protein fraction derived from leaves of *Carissa carandas* aganist *Chromobacterium violaceum* CV026, the QS signaling detected reporter strain. The results indicated that the isolated protein might influence the signaling molecule involved in the mixed bacterial biofilm. Since the fact that the QS regulated reporter phenotypes are often co-dependent on other factors, further experimental studies will validate this current finding. In general, the identification of QS signals or QSI is an interesting issue, however, adequate control experiments are essential to confirm that the compounds really possess QSI activity or QS function. For example, Levipan et al. release a commentary on the recently published study titled “*Piscirickettsia salmonis Produces a N-Acetyl-L-Homoserine Lactone as a Bacterial Quorum Sensing System-Related Molecule*” in Frontiers in Cellular and Infection Microbiology, and think that scientific knowledge regarding molecular signalling pathways to control cell density-dependent phenotypes is still in its infancy.

In summary, the work reported here reinforces the already known application of QS and QSI in agriculture, healthcare and environment. We hope that this Research Topic will initiate new studies in this exciting area where the detailed or acquired molecular mechanisms remain unexplored.

## Author contributions

LC and ML edited the Research Topic of Advances in Bacterial Quorum Sensing System and Quenching, LC, LK and ML wrote the manuscript. All authors contributed to the article and approved the submitted version.

## Funding

This work was supported in part by Natural Science Foundation of Shaanxi Province (Grant number: 2019JM-372) and the funding of the Fourth Military Medical University (No. 2018RCFC06, No. 2021XB045).

## Conflict of interest

The authors declare that the research was conducted in the absence of any commercial or financial relationships that could be construed as a potential conflict of interest.

## Publisher’s note

All claims expressed in this article are solely those of the authors and do not necessarily represent those of their affiliated organizations, or those of the publisher, the editors and the reviewers. Any product that may be evaluated in this article, or claim that may be made by its manufacturer, is not guaranteed or endorsed by the publisher.
